# A multi-country study of intussusception in children under 2 years of age in Latin America: analysis of prospective surveillance data

**DOI:** 10.1186/1471-230X-13-95

**Published:** 2013-05-27

**Authors:** Xavier Sáez-Llorens, F Raúl Velázquez, Pio Lopez, Felix Espinoza, Alexandre C Linhares, Hector Abate, Ernesto Nuñez, Guillermo Venegas, Rodrigo Vergara, Ana L Jimenez, Maribel Rivera, Carlos Aranza, Vesta Richardson, Mercedes Macias-Parra, Guillermo Ruiz Palacios, Luis Rivera, Eduardo Ortega-Barria, Yolanda Cervantes, Ricardo Rüttimann, Pilar Rubio, Camilo J Acosta, Claire Newbern, Thomas Verstraeten, Thomas Breuer

**Affiliations:** 1 Hospital del Niño, Infectious Disease Department, Avenida Balboa, Calle 34, Ciudad de Panamá, Panama; 2 Instituto Mexicano del Seguro Social, Medical Research Unit on Infectious Diseases, CMN-SXXI, Av. Cuauhtemoc 330, CP 06720 Mexico City, Mexico; 3 Centro de Estudios en Infectologia Pediatrica, Clinica Materno Infantil Los Farallones, Calle 9 C-50 #25, Piso11, Cali, Colombia; 4 Universidad Nacional Autónoma de Nicaragua (UNAN), Edificio Central, Contiguo a Ig. La Merced, Apartado Postal 68, Leόn, Nicaragua; 5 Instituto Evandro Chagas, Secretaria de Vigilância em Saúde, Virology Section, Av. Almirante Barroso 492, Belém, Pará, 66.090-000, Brazil; 6 Hospital Dr. Humberto Notti, Avda. Bandera de los Andes 2603 (CP: 5500) Villa Nueva de Guaymallén, Mendoza, Argentina; 7 Universidad de Concepción, Urrutia Manzano, Concepción, 330, Chile; 8 Escuela de Medicina, Universidad de Valparaíso, Hontaneda 2653, Oficina 318, Valparaíso, Chile; 9 Hospital Nacional de Niños, Paseo Colón, San José, Costa Rica; 10 Organización para el Desarrollo y la Investigación Salud en Honduras (ODISH), Colonia Humuya, Sendero Pastizal, número 2449, Tegucigalpa, Honduras; 11 Hospital General de Tlanepantla “Valle Ceylán”, Calle Villahermosa y Colima s/n, Tlanepantla, CP 54150, Mexico; 12 Hospital Infantil de Mexico, Calle Dr. Márquez # 162, Col. Doctores, México DF, Mexico; 13 Instituto Nacional de Pediatría, Insurgentes Sur, Col. Cuicuilco 4º piso, México DF, 3700-C, Mexico; 14 Instituto Nacional de Ciencias Médicas y Nutrición Salvador Zubirán, Vasco de Quiroga 15, Col. Sécción XVI, Tlalpan, Mexico DF, 14000, Mexico; 15 Hospital Maternidad Nuestra Sra de la Altagracia, Av. Pedro Henríquez Ureña, No.49, Gazcue, Santo Domingo, DN, República Dominicana; 16 Fundación para el Avance de la Investigación Clínica y Translacional, Consultorios Médicos América, Suite No. 727, Vía España, Carrasquilla, Panama; 17 GlaxoSmithKline Vaccines México, Calzada Mécico Xochimilco No. 4900, Colonia San Lorenzo Huipulco, Delegación Tlalpan, México DF, CP 14370, Mexico; 18 GlaxoSmithKline Vaccines Argentina, Carlos Casares 3690, Victoria, Buenos Aires, B1644 BCD, Argentina; 19 GlaxoSmithKline Vaccines Costa Rica, 400 metros Oeste de la Rotonda de la Bandera, Sabanilla, Montes de Oca, PO Box 10196-1000, San Jose, Costa Rica; 20 GlaxoSmithKline Vaccines, Philadelphia, USA; 21 GlaxoSmithKline Vaccines, Rue de I'Institut 89, Rixensart, 1330, Belgium; 22 Current address: Mexican Ministry of Health, National Center for Child and Adolescent Health, Colonia Merced Gomez, Mexico DF, Mexico; 23 Current address: GlaxoSmithKline Vaccines, Ciudad del Saber, Edifício 230, Clayton, Panama; 24 Current address: Independent Medical Professional, Buenos Aires, Argentina; 25 Current address: Merck & Co., Global Health Outcomes Vaccines, Philadelphia, PA, USA; 26 Current address: Philadelphia Department of Public Health, Philadelphia, USA; 27 Current address: P95 Excellence in Pharmacovigilance and Epidemiology, Koning Leopold III laan 1, Leuven, 3001, Belgium; 28 Current address: GlaxoSmithKline Vaccines, Parc de la Noire Epine, Rue Fleming, Wavre, 20 1300, Belgium

**Keywords:** Intussusception, Latin America, Vaccination, Rotavirus, Multi country, Children

## Abstract

**Background:**

Intussusception (IS) is a form of acute intestinal obstruction that occurs mainly in infants and is usually of unknown cause. An association between IS and the first licensed rotavirus vaccine, a reassortant-tetravalent, rhesus-based rotavirus vaccine (RRV-TV), led to the withdrawal of the vaccine. New rotavirus vaccines have now been developed and extensively studied for their potential association with IS. This study aimed to describe the epidemiology and to estimate the incidence of IS in Latin American infants prior to new vaccine introduction.

**Methods:**

Children under 2 years of age representing potential IS cases were enrolled in 16 centers in 11 Latin American countries from January 2003 to May 2005. IS cases were classified as definite, probable, possible or suspected as stated on the Brighton Collaboration Working Group guidelines.

**Results:**

From 517 potential cases identified, 476 (92%) cases were classified as definite, 21 probable, 10 possible and 10 suspected for intussusception. Among the 476 definite IS cases, the median age at presentation was 6.4 months with 89% of cases aged <1 year. The male to female ratio was 1.5:1. The incidence of definite IS per 100,000 subject-years ranged from 1.9 in Brazil to 62.4 in Argentina for children <2 years of age, and from 3.8 in Brazil to 105.3 in Argentina for children aged <1 year. Median hospital stay was 4 days with a high prevalence of surgery as the primary treatment (65%). Most cases (88%) made a complete recovery, but 13 (3%) died. No clear seasonal pattern of IS cases emerged.

**Conclusions:**

This study describes the epidemiology and estimates the incidence of IS in Latin American infants prior to the introduction of new rotavirus vaccines. The incidence of IS was found to vary between different countries, as observed in previous studies.

**Trial registration:**

Clinical study identifier 999910/204 (SERO-EPI-IS-204)

## Background

Intussusception (IS) is a form of acute intestinal obstruction that occurs mainly in infants [1]. It is the most frequent cause of acute abdominal emergency in the first two years of life but rarely occurs in adults [1]. Most cases of IS are considered idiopathic although several authors have suggested links with various infectious agents [2-4]. An association between natural rotavirus (RV) infection and IS has not been found [3,5,6]. The main argument against this association is the seasonality of RV disease in children between 3–24 months without any similar variability in IS prevalence in the same age group [6-10].

The first RV vaccine licensed in the United States (*RotaShield*™, Wyeth-Lederle) was withdrawn in 1999 because of concerns about the association of vaccination with IS [11,12]. In retrospective investigations, administration of this tetravalent rhesus human reassortant RV vaccine (RRV-TV) was associated with a significantly increased risk of IS (case–control analysis, adjusted odds ratio: 21.7; 95% CI: 9.6 to 48.9), especially in the two weeks following the first vaccination dose [5,13].

Development of a safe and effective RV vaccine has been given high priority by the WHO because of the considerable RV disease burden especially in developing countries [14,15]. Newly licensed RV vaccines include a human RV vaccine (HRV) (*Rotarix*™_,_ GlaxoSmithKline Vaccines) and a human-bovine reassortant vaccine (*RotaTeq*™, Merck & Co.). Results of large-scale, randomized, double blind, placebo-controlled studies, each involving more than 60,000 infants, show these new RV vaccines to be immunogenic and highly effective for the prevention of RV gastroenteritis and associated hospitalizations during the first year of life. Importantly, both vaccines appear to have a favorable safety profile, with both studies excluding any immediate risk of IS (risk <1 in 20,000) [16,17].

Universal RV vaccination has been introduced in many Latin American countries; since 2006, 14 countries and one territory have incorporated vaccines in their National Immunization Programs, including 12 countries using *Rotarix*™ and 2 using *RotaTeq*™ [18]. Recent post-licensure IS surveillance studies have associated rotavirus vaccination with an increased, short-term risk of IS within 7 days following the first vaccine dose among Mexican infants [19,20]. However, a recent analysis involving 14 Latin American countries using rotavirus vaccines provided evidence that health benefits of vaccination far outweigh this transient risk and support continuous RV vaccination in Latin America [21].

This study was conducted to provide an understanding of the epidemiology and to estimate the incidence of IS in Latin American infants prior to RV vaccine introduction. Since there is a need for continuous safety surveillance in countries that have adopted RV vaccination into their public sectors, our study will provide useful baseline IS rates regarding further assessment of the potential IS risk versus health benefits in Latin America.

## Methods

### Study design

This is a prospective, cross-sectional, observational study conducted between January 2003 and May 2005 at 14 centers in Argentina, Brazil, Chile, Colombia, the Dominican Republic, Honduras, Mexico, Nicaragua, and Panama. During the first year, the study was also conducted in 1 center in Peru and 1 center in Costa Rica (16 centers in total). Centers were selected by their importance as regional/national reference hospitals within the public health-care system of the above mentioned countries, capacity to perform surveillance for IS and their potential to participate in a large safety and efficacy trial of RIX4414 vaccine (*Rotarix*™, GlaxoSmithKline Vaccines, NCT00140673). The surveillance ran in parallel to the safety and efficacy trial. The study was approved by the following Institutional Review Boards at all participating centers: Comité de Ética, Hospital Humberto Noti (Argentina); Comitê de Ética em Pesquisa, Instituto Evandro Chagas, FUNASA and Comissão Nacional de Ética em Pesquisa (CONEP) (Brazil); Comité de Ética Ad Hoc, Ministerio de Salud (Chile); Unidad de Bioética e Investigación, Comité Ético Científico, Hospital de Niños Dr. Carlos Saenz Herrera, Centro de Ciencias Médicas CCSS (Costa Rica); Comité de Ética en Investigación Biomédica de la Universidad Nacional Autónoma de Honduras, Facultad de Ciencias Médicas (Honduras); Centro de Investigación y Ética del Hospital General de Tlanepantla (Mexico), Comité de Etica del Hospital del Niño Morelense, Comité de Etica del Instituto Nacional del Pediatría Insurgentes Sur, Comité de Ética e Investigación del Hospital General de Durango and Comité Local de Investigación y de Bioética Hospital General de Pediatria (Mexico); Comité de Ética para Investigaciónes Biomédicas, Universidad Nacional Autónoma de Nicaragua, Facultad de Ciencias Médicas (Nicaragua); Instituto Conmemorativo Gorgas de Estudios de la Salud Justo Arosemena (Panama), Instituto Nacional de Salud (Peru), Comité de Bioética, Hospital Maternidad Nuestra Señora de Altagracia (Dominican Republic); and Comité de Etica Clínica Materno Infantil Los Farallones (Colombia). The study was conducted in accordance with the Declaration of Helsinki, Good Clinical Practice guidelines and the International guidelines for Ethical Review of Epidemiological Studies. Written informed consent was obtained from the parents/guardians of all children prior to enrolment.

### Study population

Children receiving care for potential IS at participating hospitals were identified by systematic reviews of hospital daily records in various departments (admission, emergency department, pediatric ward, surgical department, and radiology). A screening sheet was used in all appropriate departments to identify all eligible cases. Eligibility criteria for the study were limited to: children under 24 months of age at onset of a potential IS episode; episodes that occurred during the study period; children for whom consent could be obtained from a parent or guardian; and children with no previous radiographically or surgically confirmed IS episodes. When potential IS cases that appeared to meet the eligibility criteria were identified, the child’s physician was asked for permission to discuss the study with the child’s parents/guardians. If the parents/guardians were interested in participation, written informed consent was sought.

### Data collection

Previous medical history, physical examination findings on admission, radiographic and surgical procedures performed, diagnosis, other treatments, pathologic findings, duration of hospital stay, and outcome were entered into a standardized data collection tool from medical chart review and parental/guardian interview.

### Case definition

IS cases were classified as definite, probable, possible or suspected based on the Brighton Collaboration Working Group guidelines [22,23]. According to these guidelines, the evidence for definite IS requires a) the demonstration of invagination of the intestine at surgery or b) by either gas or liquid enema, or the demonstration of an intra-abdominal mass by abdominal ultrasound with specific characteristic features that is proven to be reduced by hydrostatic enema on post-reduction ultrasound or c) the demonstration of invagination of the intestine at autopsy. If definite criteria is not fulfilled, cases were classified as probable (2 major criteria or one major criteria and 3 minor criteria); possible (4 or more minor criteria), and suspected IS represented by cases that did not meet any of the three 3 levels of evidence (definite, probable, and possible) (Table 1).

**Table 1:** Major and minor criteria used in the case definition for the diagnosis of intussusception

**1. Evidence of intestinal obstruction:**	**Predisposing factors:**
I. History of bile-stained vomiting and either	Age <1 year and male sex
II. Examination findings of acute abdominal distension and abnormal or absent bowel sounds	Abdominal pain
or	Vomiting
III. Plain abdominal radiograph showing fluid levels and dilated bowel loops.	Lethargy
**2. Features of intestinal invagination: One or more of the following:**	Pallor
I. Abdominal mass	Hypovolemic shock;
III. Rectal mass	Plain abdominal radiograph showing an abnormal but non-specific bowel gas pattern
IIII. Intestinal prolapse	
IV. Plain abdominal radiograph showing a visible intussusceptum or soft tissue mass	
V. Abdominal ultrasound showing a visible intussusceptum or soft tissue mass	
VI. Abdominal CT scan showing a visible intussusceptum or soft tissue mass.	
3. Evidence of intestinal vascular compromise or venous congestion:	
I. Passage of blood per rectum	
or	
II. Passage of a stool containing “red currant jelly” material	
or	
III. Blood detected on rectal examination	

J.E. Bines http://www.sciencedirect.com/science/article/pii/S0264410X03006637, K.S. Kohl, J. Forster, et al., Vaccine. 22 (2004) 569–574.

### Data analysis

The complete analysis included all subjects enrolled during the study duration. Following initial description of identified cases, statistical analyses were limited to definite IS cases.

For each country, the annual incidence of definite IS was calculated with 95% confidence intervals (CI) for children under 2 years and under 1 year of age. The numerator was the number of cases of definite IS which occurred in subjects living in the study area treated at a participating hospital and enrolled in the study. Study area denominators (the number of subjects living in the study area that would be expected to visit a participating hospital if they developed suspected IS) were estimated from national demographic statistics based on the most recent national census. The study area was pre-defined in accordance to the area of coverage of the participating hospitals to the district level based on the political division of the respective country. Analyses were performed using SAS® software (version 8.2).

## Results

### Study population

**Figure 1:**
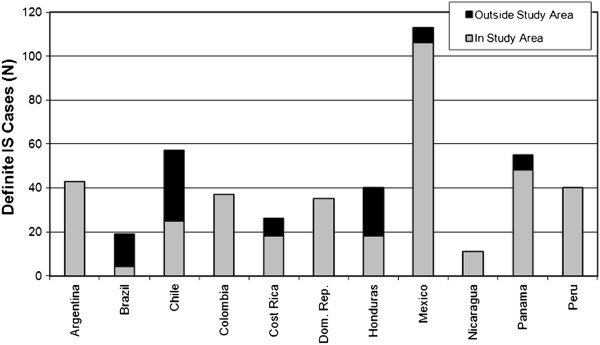
**Enrolled definite IS cases by country and study area residence (N = 476).** During the study period, 517 potential IS cases in children under 2 years of age were identified. Mexico had the highest number of definite IS case reports (n = 113; 24%).

**Figure 2:**
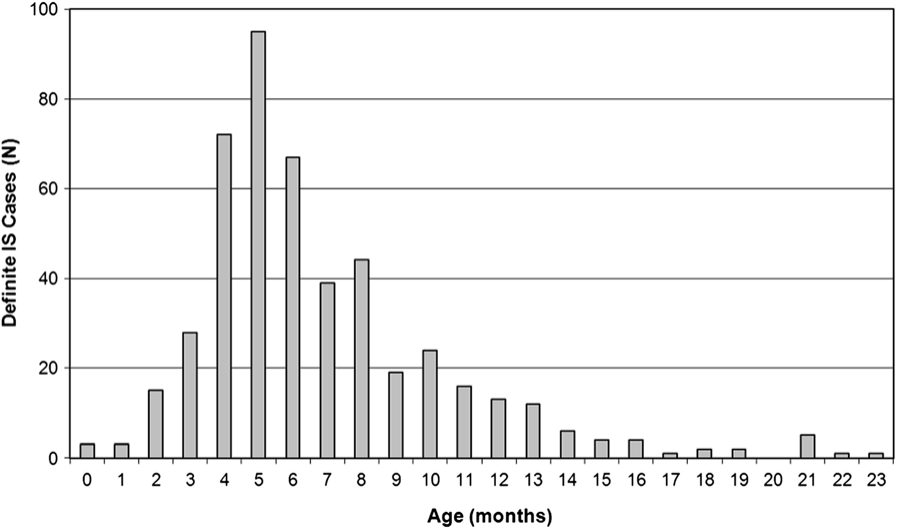
**Overall age distribution of definite IS cases in Latin American infants (N = 476).** IS cases presentation, (89%) occurred in the first year of life with a peak of cases between 4 and 8 months of age, and the highest number of cases seen among children aged 5 months.

**Figure 3:**
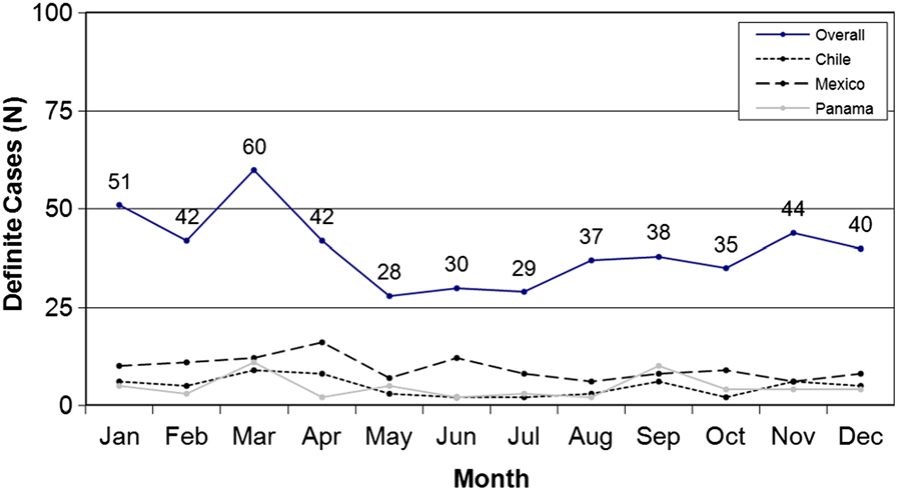
**Monthly distribution of definite IS cases (overall and in countries with >50 cases).** Distribution of cases among months of the year, no clear seasonal pattern of cases emerged either overall or within individual countries with over 50 cases.

During the study period, 517 potential IS cases in children under 2 years of age were identified. These were classified as definite (n = 476; 92%), probable (n = 21; 4%), possible (n = 10; 2%), and suspected (n = 10; 2%). Mexico had the highest number of definite IS case reports (n = 113; 24%) (Figure 1). Other countries with high number of definite cases of IS reported were Chile (n = 57; 12%) and Panama (n = 55; 12%). Reports of male cases were more common (gender ratio 1.5:1). The median age of definite IS cases at presentation was 6.4 months. Most cases (89%) occurred in the first year of life (Figure 2) with a peak of cases between 4 and 8 months of age, and the highest number of cases seen among children aged 5 months. No clear seasonal pattern of cases emerged either overall or within individual countries with over 50 cases (Figure 3).

### Definite IS incidence

**Figure 4:**
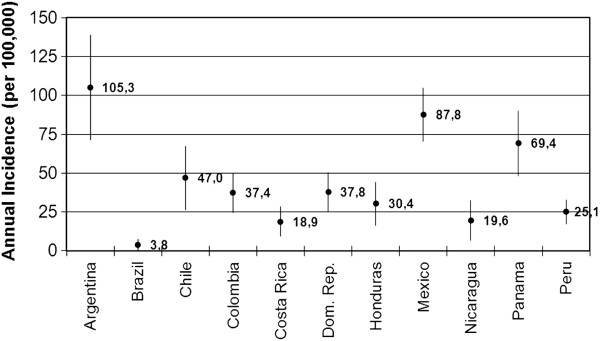
**Annual incidence of definite IS in children under 1 year of age per 100,000 subjects.** Lines indicate 95% confidence intervals. Figure shows that annual incidence of definite IS in children under 1 year of age ranged from 3.8 per 100,000 subjects in Brazil to 105.3 per 100,000 subjects in Argentina, with a wide variation in incidence.

When limited to definite IS cases who lived in the study area (n = 385; 81%), the annual incidence of definite IS for children under 2 years of age ranged from 1.9 per 100,000 subjects in Brazil to 62.4 per 100,000 subjects in Argentina, with a wide variation in incidence. The annual incidence of definite IS in children under 1 year of age ranged from 3.8 per 100,000 subjects in Brazil to 105.3 per 100,000 subjects in Argentina, with a wide variation in incidence (Figure 4).

### Clinical findings

The median hospital stay was 4 days (range 0–106 days). The most common symptoms noted on admission of definite IS cases were vomiting (91%), abdominal pain (86%), and bloody stools (75%). Other commonly reported signs and symptoms included pallor (65%), abdominal distension (60%), abnormal or absent bowel sounds (56%), bile-stained vomiting (54%), red jelly stool (54%), abdominal mass (51%), lethargy (50%), fever (49%), and blood on rectal examination (48%); 92% of cases presented with five or more symptoms.

Surgery was the most common primary treatment for children with definite IS (308 [65%] primary surgeries). Of the 168 children undergoing conservative primary treatment (ultrasound-guided gas or liquid-contrast enema), 76 (45.5%) did not undergo secondary surgery. In most (n = 66) of these cases an intra-abdominal mass with specific characteristic features, as reported in the Brighton Collaboration Group guidelines, was exhibited by abdominal ultrasound and proved to be reduced by hydrostatic enema on post-reduction ultrasound. Overall, surgery was performed on 400 (84%) children with definite IS including bowel resection in 99 (25%) cases. Invagination was identified in 84% of patients undergoing surgery with the most common involvement being ileo-cecal (89%).

Known predisposing factors were found in only 21 cases (4.4%), Meckel’s diverticulum was present in 13 cases (2.7%) and appendicitis was identified in 8 cases (1.7%) at the time of surgery. A total of 130 (27.3%) subjects had an upper respiratory tract infection in the 2 weeks before the onset of definite IS. Sixty-three subjects (13.2%) reported an illness other than upper respiratory tract infection during this period of time. The remaining 283 subjects (59.5%) did not report any illness within the 2 weeks before onset of definite IS.

### Laboratory findings

A stool sample was obtained from 99 (21%) of the definite IS cases. Various pathogens were tested for, yielding 15 positive results for: adenovirus (1 out of 3 tests), rotavirus (4 out of 43 tests), *Escherichia coli* (6 out of 23 tests), *Shigella* (1 out of 22 tests), *Cryptosporidium* (1 out of 2 tests), *Entamoeba histolytica* (1 out of 8 tests) and other unspecified parasites (1 out of 6 tests). All 7 tests for Campylobacter and 21 tests for Salmonella were negative.

### Patient outcome

Of the 476 definite IS cases, 418 (87.8%) recovered without sequelae, 42 (8.8%) recovered with sequelae, 13 (2.7%) died, and 3 (0.7%) were transferred to other hospitals and were lost to follow-up.

## Discussion

This study estimated the incidence of definite IS in young children in 11 Latin American countries as 1.9 to 62.4 per 100,000 children under 2 years of age and 3.8 to 105.3 per 100,000 children under 1 year of age, with a wide variation in incidence. The estimates for children under 1 year of age are in a similar range to published estimates from other regions, 38 per 100,000 in Switzerland (study period: 2003–2006) [24], 65 per 100,000 in New Zealand (study period: 1998–2003) [10], 66 per 100,000 in the UK [25], 71 per 100,000 in Denmark (study period: 1990–2001) [26] and 78–100 per 100,000 in Hong Kong (study period: 1997–1999). [8] However, the increase in definite IS incidence in the second 6 months of life seen in other studies [24,26-28], was not demonstrated in this study.

Our estimated IS incidence rates were similar to those previously reported from this region. In a 5-year retrospective study in Panama (study period: 1998–2002), annual rates of 19 to 40 cases of IS per 100,000 children under 1 year of age, and 8 to 15 per 100,000 in children under 3 years of age were reported depending on the year of the study [29]. A study in Chile estimated the annual IS rates in children under 1 year and under 2 years of age to be 47–55 per 100,000 and 32–35 per 100,000, respectively [30]. A study conducted in Venezuela reported an annual IS-related hospitalization rate of 35 per 100,000 in infants under 1 year of age [31]. In Brazil, where this study showed the lowest incidences of definite IS despite case identification efforts that were equally intensive as those in other participating countries, the World Health Organization has reported similarly low annual incidence (3.5 per 100,000 children under 1 year of age) [32]. There seems to be no clear explanations for the variability in observed IS rates between different Latin American countries, although intussusception incidence can vary by region [32]. Country-specific incidence rates do not seem to suggest ethnic or geographic influences (e.g. a North–south gradient). While differences in healthcare access and patient management (i.e., in some areas IS cases could not have been seen by medical personnel) are likely to play a role, other unknown factors that may be linked to IS, such as genetic influence, cultural differences, infectious diseases and gastrointestinal infections, may also be involved [11,33-35]. It should be pointed out however that, when defining specific catchment areas, it was assumed that most of potential IS cases would reach the sentinel hospitals for proper treatment, where intensive, daily surveillance was carried out. It is however recognized that less control could be exerted over cases that might come from outside the study areas.

We found a relatively high proportion of subjects (27.3%) who experienced IS to have had an upper respiratory tract infection in the 2 weeks before the onset of definite IS. The condition is known to be associated with a number of childhood viral illnesses [33], in particular with adenovirus [34], and a link with respiratory syncytial virus (RSV) infection has recently been reported [35].

The epidemiologic characteristics of definite IS cases in this study were similar to those found in other locations or previously in these countries. The majority of definite IS cases occurred before 1 year of age with a peak around 4–6 months of age, which is similar to that observed in the US [36] and other countries [24,25,29,30]. The excess of male cases has been noted in other studies with male:female ratios of 1.8:1 and 1.4:1 reported from Panama and the US, respectively [29,36].

The proportion of children undergoing surgery in the present study (84%) was similar to other Latin American studies, which have reported rates of 78% and 88% in Chile and Venezuela, respectively [30,31,37]. However, these rates are much higher than those reported from Hong Kong (23%) and the US (53%) [8,9]. Higher surgery rates in Latin America may be due to delays in seeking medical attention, more severe episodes, or less experience in radiological reduction techniques. Moreover, it seems likely that IS cases that resolved spontaneously were not included, thus possibly leading to an underestimate of the overall rate. The typical clinical presentation (vomiting, pain or irritability, and bloody stools) found in this study is similar to other reports as was the low rate of predisposing conditions, such as Meckel’s diverticulum [30-32,38]. Of interest, among the 465 subjects for whom the type of vaccine previously administered was known, 27 (5.8%) had received human rotavirus vaccine or placebo because they were participating in an ongoing phase III clinical trial in Latin America. Nevertheless, it seems very unlikely that such a low percentage might have influenced our overall estimates for the occurrence of IS prior to vaccine introduction.

The present study, like several others including some from Latin America, showed no clear seasonality of IS-associated hospitalisations [6,29,31]. The distribution of definite IS cases did not correspond with the seasonality of RV in any of these countries. As in other regions, the occurrence of RV is higher in winter months in Latin America, however, the timing and duration varies by geographical location with peaks generally occurring in Mexico between November and April [6,39], in Argentina between April and August, in Brazil between May and July, and in Chile between June and September [40,41].

We acknowledge the potential limitations of our study. To estimate the incidence rates, we relied on census data as denominators. The reliability of these census data is variable. Whereas these data may be valid on national or regional levels, they may be less precise for smaller regions such as those that defined the catchment areas for some centers. It is likely that this uncertainty accounts for some of the difference between the estimated incidence rates in different countries. We excluded children who lived outside the defined study areas. However, study area boundaries may not have had the same level of precision for all hospitals, which could affect the incidence rates presented. It is therefore likely that some cases of definite IS may have been referred to sentinel hospitals from neighborhood localities that were surrounding the originally defined study area. Also, in most countries, the study took place in a limited number of regional/national reference hospitals and therefore the incidence rates for each country cannot be regarded as national estimates.

It is possible that some of the cases that occurred in the population counted as denominator may have been treated in another hospital not involved in the study, in particular private institutions. Although a systematic review of private institutions within the study area was also performed during the surveillance and efficacy trial. To ensure that the study procedures conformed across all investigator centers add to limit potential under-reporting, the study protocol, case report form and safety reporting were reviewed with all investigators and other personnel responsible for the conduct of the study prior to study start and at regular interval periods. Adherence to the protocol requirements and verification of data generation accuracy was achieved through monitoring visits to each investigator site. Furthermore, computer checks and blinded review of subject tabulations were performed to ensure consistency of case report form completion. Finally, some parents (n = 22) refused participation. As we did not know the age of these children, we could not adjust our incidence rates to account for these children, which may have resulted in an underestimation.

## Conclusions

Our findings are in line with previously reported figures and suggest substantial variation in the rates of IS across Latin America. This study provides a recent estimate of the incidence of IS in Latin American infants, which is helpful in monitoring the safety of new RV vaccines following their introduction in the region. A few of these previously foreseen post-licensure evaluations have recently detected a transient, 4 to 6-fold increased relative risk of IS within 7 days following first rotavirus vaccine dose in Mexico. Nevertheless, recent estimates have shown that hospitalisations and deaths averted as a result of vaccination far outweigh the number of possible vaccine-related IS cases and health authorities still continue to strongly recommend routine rotavirus vaccination of infants in Latin America.

## Abbreviations

HRV: Human RV vaccine; IS: Intussusception; RRV-TV: Reassortant-tetravalent, rhesus-based rotavirus vaccine; RV: Rotavirus; WHO: World Health Organization.

## Competing interests

This study and all costs associated with the development and the publishing of the present manuscript were funded by GlaxoSmithKline (GSK) Biologicals SA. XSLL, FRV, PL, FE, ACL, HA, EN, GV, RV, ALJ, MR, CA, VR, MMP, GRP, LR and EOB were funded through their institutions by GSK. YC, RR, PR, CJA, CN, TV and TB were employed by GSK at the time of this study. EOB, YC, PR and TB are GSK employees.

## Authors’ contributions

YC, RR, PR, CJA, CN, TV and TB contributed to conception and design of the study. XSLL, FRV, PL, FE, ACL, HA, EN, GV, RV, ALJ, MR, CA, VR, MMP, GRP, LR and EOB contributed to subject recruitment and to the analysis and interpretation of the data. All authors were involved in the critical revision of drafts and have approved the final manuscript version for submission.

## Pre-publication history

The pre-publication history for this paper can be accessed here:

http://www.biomedcentral.com/1471-230X/13/95/prepub

## Trademark

*Rotarix* is a trademark of GlaxoSmithKline group of companies.

*RotaTeq* is a registered trademark of Merck & Co.

*Rotashield* is a trademark of Wyeth-Lederle, USA.
